# Feasibility of using two‐dimensional array dosimeter for *in vivo* dose reconstruction via transit dosimetry

**DOI:** 10.1120/jacmp.v12i3.3370

**Published:** 2011-04-08

**Authors:** Heeteak Chung, Jonathan Li, Sanjiv Samant

**Affiliations:** ^1^ Department of Nuclear and Radiological Engineering University of Florida Gainesville FL USA; ^2^ Department of Radiation Oncology University of Florida Gainesville FL USA

**Keywords:** *in vivo* dose reconstruction, 2D dosimeter, transit dose

## Abstract

Two‐dimensional array dosimeters are commonly used to perform pretreatment quality assurance procedures, which makes them highly desirable for measuring transit fluences for *in vivo* dose reconstruction. The purpose of this study was to determine if an *in vivo* dose reconstruction via transit dosimetry using a 2D array dosimeter was possible. To test the accuracy of measuring transit dose distribution using a 2D array dosimeter, we evaluated it against the measurements made using ionization chamber and radiochromic film (RCF) profiles for various air gap distances (distance from the exit side of the solid water slabs to the detector distance; 0 cm, 30 cm, 40 cm, 50 cm, and 60 cm) and solid water slab thicknesses (10 cm and 20 cm). The backprojection dose reconstruction algorithm was described and evaluated. The agreement between the ionization chamber and RCF profiles for the transit dose distribution measurements ranged from ‐0.2%~ 4.0% (average 1.79%). Using the backprojection dose reconstruction algorithm, we found that, of the six conformal fields, four had a 100% gamma index passing rate (3%/3 mm gamma index criteria), and two had gamma index passing rates of 99.4% and 99.6%. Of the five IMRT fields, three had a 100% gamma index passing rate, and two had gamma index passing rates of 99.6% and 98.8%. It was found that a 2D array dosimeter could be used for backprojection dose reconstruction for *in vivo* dosimetry.

PACS number: 87.55.N‐

## I. INTRODUCTION

Intensity‐modulated radiation therapy (IMRT) allows the dose distribution to be shaped to the target while avoiding normal structures by using an intensity‐modulated beam produced with a multileaf collimator (MLC). The IMRT beam‐delivery system generally produces a dose distribution that contains high‐gradient regions, which can potentially pose errors during beam delivery. Therefore, pretreatment verification of the IMRT delivery process has become a routine practice. It begins by verifying the IMRT plan using films^(^ ^1^ ^,^ ^2^ ^)^ and ionization chambers^(^ ^3^ ^–^ ^5^ ^)^ to evaluate the mechanical delivery system (e.g., beam output or MLC movement) with a phantom (i.e., solid water slab).

Although useful, pretreatment IMRT verification based on film and ionization chamber measurements is a labor‐intensive task that generates limited real‐time information. Others have demonstrated pretreatment IMRT verification using an electronic portal imaging device (EPID).^(^ ^6^ ^–^ ^11^ ^)^ While relative dosimetry is feasible, absolute dosimetry using EPID requires labor‐intensive corrections.^(6)^ To streamline the pretreatment IMRT verification process, two‐dimensional (2D) array dosimeters were developed to overcome these inherent limitations. Two‐dimensional array dosimeters perform the same function as films and ionization chambers and EPIDs for dosimetry, but they offer straightforward absolute‐dose measurements, easy setup, and instant dose‐measurement feedback and evaluation.

Three commercially available 2D array dosimeters are currently widely used for IMRT pretreatment verification: MapCHECK Model 1175 (Sun Nuclear, Melbourne, FL, USA),^(^ ^12^ ^,^ ^13^ ^)^ MatriXX (IBA Dosimetry, Schwrazenbruck, Germany),^(^ ^14^ ^–^ ^18^ ^)^ and 2D‐ARRAY seven29 (PTW‐Freiburg, Germany).^(^ ^19^ ^,^ ^20^ ^)^ Many researchers have reported highly favorable responses with proper calibration and setup of these devices. For instance, Létourneau et al.^(13)^ and Jursinic and Nelms^(12)^ reported that the MapCHECK had a linear dose response and that all diodes were calibrated to within ± 1% of each other (mostly within ± 0.5%). They also reported that MapCHECK readings were reproducible to within a maximum standard deviation (SD) of ± 0.15%, and noted a temperature dependence of 0.57%/°C, which should be taken into account for absolute dosimetric measurement. Both Spezi et al.^(17)^ and Herzen et al.^(15)^ showed 1% discrepancy when compared against an ionization chamber using MatriXX 2D array dosimeter. And lastly, Poppe et al.^(20)^ reported 2D‐ARRAY dosimeter could detect a 2 mm MLC misalignment in terms of dose difference of 5% to 15%, when compared to the non‐misaligned MLC.

Although pretreatment verification of IMRT delivery is important for verifying the mechanics and output of the linear accelerator, it does not verify all aspects of the delivery process. The biggest limitation of how IMRT verification is currently performed is that it is only done before treatment. In most clinical settings, once this pretreatment IMRT verification is done, no additional verification (or any other significant monitoring of the dose delivered to the patient) is performed. Researchers have investigated the use of diodes for *in vivo* dosimetry during treatment and have found that diode‐based *in vivo* dosimetry yields an agreement to within ± 10% of the planned radiation dose.^(^ ^21^ ^–^ ^23^ ^)^ However, diode‐based *in vivo* dosimetry usually involves a single‐point measurement at the central axis, and does not verify the complex fluence modulations associated with IMRT beams.^(^ ^21^ ^–^ ^23^ ^)^

Organ motion^(^ ^24^ ^–^ ^27^ ^)^ or shift changes in tumor volume,^(28)^ setup errors, and patient weight loss could introduce uncertainties/errors into IMRT delivery. As mentioned above, IMRT generates tightly conformed, nonuniform beam intensities around the target with hot spots within the target volume and high‐dose gradient regions adjacent to the target volume. Consequently, uncertainties in treatment delivery can arise if the hot spots occur at the periphery of the planning target volume (PTV) where the target volume overlaps or abuts any critical structures. During the course of treatment, setup errors and/or organ motion can cause the hot spots to migrate to critical structures, potentially compromising the treatment outcome.

Because of organ motion, organ deformation and mechanical errors, the assumption that a single pretreatment verification of IMRT delivery is sufficient to adequately monitor the dose delivered to the patient is overly simplistic and does not reflect the clinical reality. Interfractional *in vivo* dose reconstruction may be the preferred method for monitoring the dose delivered to the patient while accounting for organ motion or deformation and mechanical errors. In the current state of *in vivo* dose reconstruction, radiation transmitted through the patient is detected by a dosimeter. This is generally called transit dosimetry. It is used to reconstruct the dose delivered to the patient during the treatment.^(^ ^29^ ^–^ ^54^ ^)^ The goal of evaluating the interfractional *in vivo* dose reconstruction using transit dosimetry is to verify the dose delivered to the target per fraction. *In vivo* dose reconstruction is currently done by collecting the transit fluences (transmitted radiation through the patient) with an EPID and reconstructing the dose delivered to the patient by backprojecting the detected transit fluences from the EPID to the patient volume. Reconstructing the dose via backprojection makes it possible to directly compare the reconstructed dose and the calculated dose in the patient volume. EPIDs have been heavily utilized for this type of work because of their ease of deployment, positional accuracy, multitude of measurement points, and automated signal digitization.^(^ ^35^ ^,^ ^55^ ^–^ ^63^ ^)^

The purpose of this study was to show that a 2D array dosimeter can be an alternative for EPIDs in the application of *in vivo* dose reconstruction via transit dosimetry. The current study was based on the backprojection dose reconstruction method using EPIDs by Boellaard et al.^(29)^ For this work, the measurement and accuracy of transit dose distribution using a 2D array dosimeter was evaluated against an ionization chamber and radiochromic films. A description of the backprojection dose‐reconstruction algorithm was described. Lastly, the reconstructed dose distribution was evaluated using solid‐water slabs.

## II. MATERIALS AND METHODS

### A. Linear accelerator and 2D array dosimeter

Six megavoltage photon beams were delivered using an Elekta Synergy linear accelerator (Elekta Oncology, Crawley, UK). The gantry and collimator rotation angles were set to 0°. No wedges were used. The beam profiles were scanned using a three‐dimensional (3D) water scanning system (IBA Dosimetry, Schwarzenbruck, Germany). The profile scans were made using a small ion chamber (CC04, IBA Dosimetry, Schwarzenbruck, Germany), which has a sensitive volume of 0.04 cm^3^. The water tank profile scans were used as the reference profiles.

The MapCHECK Model 1175 consists of 445 radiation‐hardened N‐type diodes that are in a $22\times 22\;{\text{cm}}^{2}$ 2D array with variable spacing between the diodes. Each detector has an active area of $0.8\times 0.8\;{\text{mm}}^{2}$. The $10\times 10\;{\text{cm}}^{2}$ center of the MapCHECK contains 221 diodes spaced 10 mm apart, and each line of detectors translates to 5 mm with respect to the next, so that the diagonal spacing between the detectors is 7.07 mm. The outer part of the MapCHECK contains 224 diodes spaced 20 mm apart; each line is shifted 1 cm and the diagonal spacing becomes 14.14 mm. Diode sensitivity was calibrated for each diode relative to the central diode using a built‐in software application with the user's linear accelerator. The dose calibration was also done using the built‐in software application to calibrate doses before each measurement.

To obtain enough sampling points to properly characterize the transit fluence, especially in the penumbra region, a motorized stepper platform (MapCHECK XY; Sun Nuclear, Melbourne, FL, USA) was used to increase the sampling of the 2D array dosimeter grid spacing. Chung et al.^(64)^ and Dempsey et al.^(65)^ showed that a 2 mm grid resolution was sufficient to avoid any significant errors larger than 1% for clinical IMRT plans. To increase the sampling resolution of the measurements, the 2D array dosimeter system was mounted on a MapCHECK XY stepper to precisely translate in the cross‐plane direction by 2 mm steps to achieve a resolution of 2 mm.

### B. Radiochromic film

In addition to a water tank profile scan, films were used to evaluate the transit fluences and reconstructed dose distributions. For the film measurements, GAFCHROMIC EBT (Industrial Specialty Products, Wayne, NJ, USA) radiochromic film (RCF) was used. RCFs are a good dosimeter for ionizing radiation dosimetry because of their near energy independence, high spatial resolution, and tissue equivalence.^(66)^ The Epson Expression 10000XL Professional flatbed document scanner with a xenon gas cold cathode fluorescent lamp and a CCD line sensor (Epson America Inc., Long Beach, CA, USA) was used to digitize the RCF. The scanner had a maximum pixel depth of 48 bits per pixel (16 bits per color channel), and the maximum read area was $21.6\times 29.7\;{\text{cm}}^{2}$. RCF irradiation and digitization were performed, as described previously.^(67)^ The calibration and measurement films were handled together to maintain similar temperatures and humidity. Small strips of film were cut $\left(5\times 5\;{\text{cm}}^{2}\right)$ from the calibration film. Each film strip was placed in the solid water slab at the linear accelerator's calibration condition and known doses were delivered. The Epson scanner was used to digitize the films. No particular bowing effect correction or significant image processing were done to the digitized films.^(68)^ The calibration films were used to generate a sensitometric curve. A fourth‐order polynomial function was fitted to the curve and was used to convert the optical density to dose.

**Figure 1:**
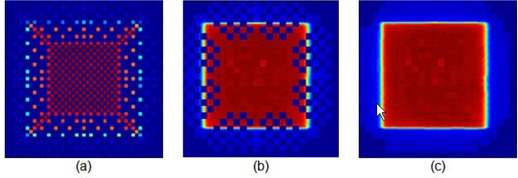
Transit dose distributions for a $10\times 10\;{\text{cm}}^{2}$ field size at 160 cm source to detector distance: (a) two‐dimensional dose distribution of MapCHECK system when irradiated without the use of motorized table; (b) MapCHECK dose distribution with the use of motorized table but without the use of GAFCHROMIC EBT film to fill in the missing data points; (c) MapCHECK dose distribution with the use of motorized table and GAFCHROMIC film to fill in the missing data points. Out of 14,400 data points, 6,175 data points were filled in using the GAFCHROMIC EBT film.

The RCFs were used to fill in some of the areas of the 2D array dosimeter where no measurements were possible because the diode detectors were manufactured in a star formation outside the $10\times 10\;{\text{cm}}^{2}$ area (Fig. 1(a). Even with the motorized stepper, it was not possible to collect all the data for all the grid spaces, especially for field sizes larger than $10\times 10\;{\text{cm}}^{2}$. (Figure 1b) shows the MapCHECK dose distribution obtained without using RCFs to fill in the missing data points. Note that even with the motorized stepper, for a $10\times 10\;{\text{cm}}^{2}$ open field at a source‐to‐detector distance (SDD) of 160 cm, there were areas where no transit fluences were sampled. Using the RCFs, the missing 2D array dose distribution was filled in for the peripheral area by irradiating the film in the same setup condition as the dosimeter and the missing data points were filled in (Fig. 1(c). This technique was applied to any 2D dose distribution with missing data points within the area of interest. Of 14,400 data points, 6,175 were filled in using RCF.

### C. Measuring the transit dose distribution

**Figure 2:**
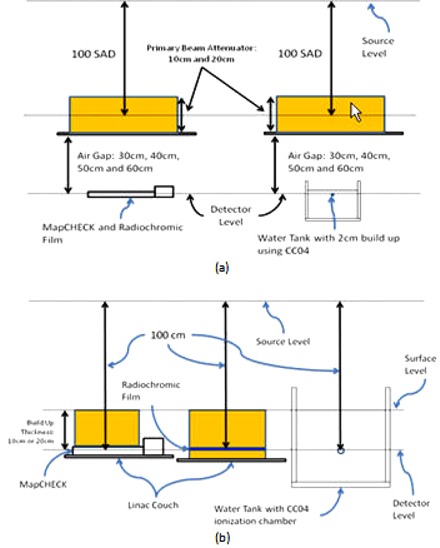
Illustration of: (a) the setup for an air gap of 30 cm, 40 cm, 50 cm, and 60 cm; (b) a setup for a 0 cm air gap. The distance from source to detector (MapCHECK, Radiochromic film, and CC04) was always kept at 100 cm. Backscatter thickness of 5 cm was used. MapCHECK has an inherent backscatter thickness of 2.7 cm of water‐equivalent material.

**Figure 3:**
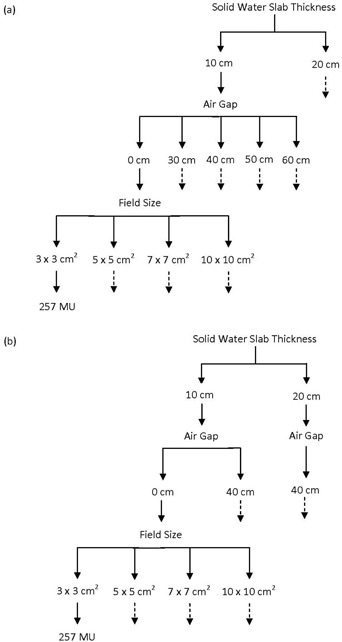
Flow chart of the transit fluence measurement parameters: (a) of the transit fluence measurement parameters using the 2D array dosimeter; (b) of the transit fluence measurement parameters using RCF. The dashed arrows indicate the same parameters as indicated by the solid arrow. MU is monitor unit.

Figure 2 shows the setup that was used to perform the transit fluence measurements. For the transit fluence measurements, the CC04 was used as the reference. RCF was used to provide an additional comparison between MapCHECK and CC04. The MapCHECK has 2 cm water‐equivalent acrylic buildup material built into the detector casing. Thus, all other dosimeters (RCF and CC04) were positioned to ensure that a total of 2 cm water‐equivalent buildup material (either solid water slabs or water) was placed on top of the CC04 and RCF (Fig. 2(a). The MapCHECK has 2.7 cm of water‐equivalent backscatter material built into the casing. Five centimeters of a backscatter material, except for the CC04, was positioned at the bottom of the RCF to ensure that a similar backscatter condition was maintained. These buildup and backscatter conditions were maintained for all measurements. (Figure 2b) shows the experimental setup for the case of 0 cm air gap. For the case of 0 cm air gap, 10 cm and 20 cm solid water slabs were used. The 2D array dosimeter and RCFs were positioned at 100 cm source‐to‐detector distances. A flow chart of the transit fluence measurement parameters is shown in Fig. 3. A single measurement was done for each parameter.

To evaluate the dosimeter and the RCF against the CC04 for a given profile, the central axis (CAX) percent dose difference and average difference were used. The CAX percent dose difference was the dose difference (CAX percent dose difference = $\left({\text{Dosimeter}}_{@\text{CAX}}-{\text{CC}04}_{@\text{CAX}})/{\text{CC}04}_{@\text{CAX}}\right)$ at the CAX of the profile normalized with the ionization chamber dose. The average difference was the difference between the CC04 and either the dosimeter or the RCFs within the 50% of the profile (see Eq. (1)). Beyond the 50% penumbra region, the profiles were not analyzed. The reasons for this were that the doses beyond 50% penumbra regions were very low and pose little significant to the overall dose, and percent dose differences would be over amplified at such low doses.

(1) $$average\_difference=\frac{1}{N}\sum_{i=1}^{N}\left(\frac{Dosimeter_{i}-CC04_{i}}{CC04_{i}}\times 100\right)$$

For IMRT 2D transit dosimetry measurements, the RCFs were used as the reference. We evaluated five clinical IMRT fields based on plans for head‐and‐neck IMRT boosts. Both the MapCHECK and the RCFs were positioned at the 50 cm air gaps. For the 0 cm air gap, the 2D array dosimeter was positioned at the CAX of the beam with a total of 10 cm of buildup material. The RCFs were positioned in the CAX of the beam with 10 cm of buildup material and 5 cm of backscattering material. The source‐to‐film distance was maintained at 100 cm. For the 50 cm air gap, both the dosimeter and the RCF had 10 cm solid water slabs to attenuate the primary beam. RCF irradiation and digitization were performed as mentioned above. The RCFs were then imported into MATLAB R2009a (The MathWorks, Natick, MA, USA) for evaluation using the gamma index test.^(69)^ The gamma index criteria were set at a 3% dose difference and a 3 mm distance‐to‐agreement (DTA) at the CAX.

### D. The backprojection dose‐reconstruction algorithm

As stated before, this study was a feasibility study to determine the usage of a 2D array dosimeter for *in vivo* dose reconstruction via transit dosimetry; it is not possible to apply the current setup to an actual patient treatment application. The 2D array dosimeter was positioned at 50 cm air gap (Fig. 2(b). Twenty‐centimeter‐thick solid water slabs were positioned at 90 cm SSD in an isocentric setup. For each field, the MapCHECK XY motorized stepper was used to generate 2D dose distributions with a grid space of 2 mm. For the IMRT fields, each segment was separately irradiated and reconstructed, and all the segments were summed up to generate the intensity‐modulated field.

#### D.1 Characterization of scatter photons from the phantom to the detector

**Figure 4:**
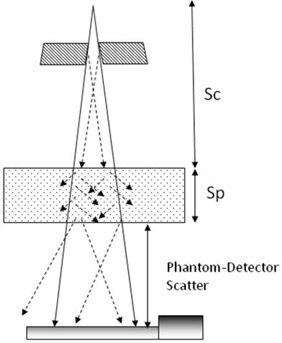
Illustration of experimental setup of transmission fluence measurement. The scatter photons are created from the source down to the exit side of the solid water slab. By the time the transit fluence is detected by the two‐dimensional (2D) array dosimeter, the majority of scatter photons generated from the solid water slabs are rejected due to the air gap. The solid arrow represents primary photons, and the dotted arrow represents scatter photons.

Figure 4 illustrates the transmission fluence measurements for the scatter photon study. Initially, as the primary photons travel from the source to the phantom, the output of the linear accelerator (Sc) consists of the primary and scatter photons. As these photons hit the phantom's surface, the scatter photons originating from the phantom (Sp) start to contribute to the total dose deposition. Thus, by the time the photons traverse and exit the phantom, the scatter fraction (ratio of scatter photons to total photons) can range from 15% to 35%, depending on the nominal beam energy, field size, and phantom thickness.^(70)^ Once the photons exit the phantom, an air gap acts as an indirect scatter rejecter. Therefore, when the photons reach the 2D array dosimeter, a higher proportion of the photons reaching the dosimeter are primary photons. Nevertheless, some scatter photons do reach the dosimeter. For the backprojection dose reconstruction to work properly, only the primary dose has to be backprojected. The scatter photons reaching the 2D array dosimeter were quantified in order to account for the scatter contribution during the dose reconstruction.

Since the scatter photons cannot be measured directly, the scatter fraction must be determined indirectly by extrapolating the transmission fraction (the ratio of transit fluence measured with and without solid water slabs) from open fields down to a zero field size. The transmission fraction at the zero field size represents the primary component. The scatter fraction was obtained by subtracting the transmission fraction of a field size of interest to the zero field size.^(^ ^70^ ^,^ ^71^ ^)^

To evaluate the scatter fraction, field sizes of $3\times 3\;{\text{cm}}^{2},5\times 5\;{\text{cm}}^{2},7\times 7\;{\text{cm}}^{2},10\times 10\;{\text{cm}}^{2}$, and $15\times 15\;{\text{cm}}^{2}$ and air gaps of 40 cm, 50 cm, and 60 cm were used. Twenty‐centimeter‐thick solid water slabs were used to simulate the patient. MapCHECK, CC04, and RCF were used for the CAX evaluation. For the profile evaluation, MapCHECK and RCF were used.

#### D.2 Four parameters for backprojection dose reconstruction

Our method for backprojection dose reconstruction was similar to that proposed by other researchers for EPIDs.^(^ ^29^ ^,^ ^39^ ^,^ ^54^ ^)^ For the dose reconstruction, the following four parameters were considered:

1) Inverse square correction factor (ISCF)
2) Attenuation correction factor (ACF)
3) Scatter correction factor (SCF), and
4) Scatter kernel (SK)

The ISCF corrects for the difference in divergence between the detector position and the dose reconstructed plane, given by:

(2) $$ISCF={\left(\frac{SDD}{SPD}\right)}^{2}$$

where SDD is the source‐to‐detector distance and SPD is the source‐to‐planar distance, which is determined by the user according to the planning computed tomography (CT) coordinate system on the CAX of the beam.

The ACF accounts for the attenuation from the detector to the user‐selected dose reconstructed plane. This correction takes the transit dose collected from the 2D array dosimeter and corrects it for the attenuation of the primary beam from the plane to the detector. The ACF is given by:

(3) $$ACF=e^{\mu }{\text{eff}}^{\cdot d}$$

where $\mu_{\text{eff}}$ is the effective linear attenuation coefficient, which can be ascertained from the planning CT data set. The physical distance, *d*, is the physical length from the 2D array dosimeter to the backprojected dose plane. Both the linear attenuation coefficient and the physical distance were obtained from a ray‐tracing algorithm.^(^ ^72^ ^,^ ^73^ ^)^ The attenuation of air is taken into consideration during this process.

The SCF accounts for the dose contribution from the scatter photons originating from the linear accelerator collimator and the phantom. This was achieved by first collecting the transit fluences for three different field sizes $(3\times 3\;{\text{cm}}^{2},5\times 5\;{\text{cm}}^{2}$, and $10\times 10\;{\text{cm}}^{2}$) for known doses. The transit dose collected by the 2D array dosimeter was backprojected, taking the ISCF and ACF into account. SCF was determined by initially subtracting the total dose to the backprojected dose (product of the transit dose collected by the 2D array dosimeter, ISCF, and ACF but not scatter components). A ratio of the subtracted dose and the total dose was obtained to determine the SCF. Both the collimator and the phantom scatters were expressed in a single factor in SCF. In practice, once the primary dose has been backprojected from the dosimeter to the depth of interest, the ray‐tracing algorithm would continue to ray‐trace from the dose reconstructed plane to the surface of the phantom proximal to the source. This procedure would determine the radiological path from the dose reconstructed plane to the phantom's surface. The radiological path would be used along with the equivalent square field size to estimate the scatter contribution of the total dose. Because the scatter contribution will vary with respect to the depth (distance from the phantom's surface to the reconstructed dose plane) and the field size, the transit fluences must be collected for each individual IMRT segment.

Finally, the SK corrects for the lateral scatter in the phantom by convolving the total dose to properly characterize the lateral scatter of the penumbra region for each segment. The scatter kernel is characterized by:

(4) $$SK=e^{-\frac{r_{ij}^{2}}{A^{2}}}+B\cdot e^{-\frac{r_{ij}^{2}}{C^{2}}}+D\cdot e^{-\frac{r_{ij}^{2}}{E^{2}}}$$

where *r* is the distance of the pixel *ij* from the CAX, and A, B, C, D, and E are the kernel parameters. The area under the kernel was normalized to unity before the convolution. The optimal scatter kernels were obtained by iteratively adjusting the five parameters so that the reconstructed dose profiles provided the best fit to the ion chamber‐measured profiles for three different field sizes (i.e., $3\times 3\;{\text{cm}}^{2},5\times 5\;{\text{cm}}^{2}$, and $10\times 10\;{\text{cm}}^{2}$) with varying depths. An interpolation was done to generate interpolated scatter‐kernel parameters appropriate to the given equivalent square segment. When all four parameters are combined, the reconstructed dose at a plane is:

(5) $$D_{reconstructed}=\left(D_{primary}\cdot ISCF\cdot ACF\cdot SCF\right)⊗SK$$

where $D_{\text{primary}}$ is the transit dose detected by the 2D array dosimeter and is the convolution operator.

### E. Evaluating the algorithm

To evaluate the algorithm, we positioned 20 cm thick solid water slabs at a 90 cm SSD and the dosimeter at 160 cm SDD (air gap of 50 cm). Three sets of clinical measurements were used in the evaluation. The first part of the evaluation consisted of generating a depth‐dose profile for a $5\times 5\;{\text{cm}}^{2}$ field size and evaluating it against the ionization chamber depth‐dose profile. A planar dose was reconstructed from a depth of 2 cm to 15 cm at increments of 1 cm. The CAX point was used for the depth‐dose profile.

The second part of the evaluation was the dose reconstruction of six irregularly shaped conformal fields at a 10 cm depth. The conformal fields were evaluated against the treatment planning system (TPS) generated planar dose distribution (Pinnacle^3^, version 8.0m, Philips Medical Systems, Madison, WI, USA). The TPS generated planar dose was used as the reference to simulate how this method would be applied in the clinical setting. All six fields were irradiated with 300 MU at a 160 cm SDD using 20 cm solid water slabs (Fig. 2). The transit fluences were collected using the 2D array dosimeter and the planar dose was reconstructed at a 10 cm depth (100 source‐to‐axis distance (SAD)) using the dose reconstruction algorithm described previously. For all fields, any missing data points were filled in using RCF. To evaluate two planar doses (i.e., dose reconstructed planar dose and TPS generated planar dose), 3%/3 mm gamma index criteria were used. The passing gamma indexes were set to be 1 or less. Any points with a gamma index exceeding 1 were considered failures.

The final part of the algorithm evaluation involved five step‐and‐shoot clinical IMRT fields. For each clinical IMRT field, there were five segments. The transit fluences were collected for each segment. All IMRT fields were set up to mimic the setup of the conformal fields (i.e., 6 MV, 160 cm SDD, and 20 cm of solid water slabs). The dose reconstruction algorithm was applied to each individual segment to reconstruct the 2D dose distribution at the 10 cm depth. All five segments were summed up to generate an intensity‐modulated dose distribution which was evaluated against the TPS generated planar dose. As in the conformal field evaluations, planar doses were used to simulate how this method would be used in a clinical setting. The IMRT fields were evaluated against the TPS generated planar dose by using the gamma criteria of 3%/3 mm.

## III. RESULTS

### A. Measuring transit dose distribution

**Figure 5:**
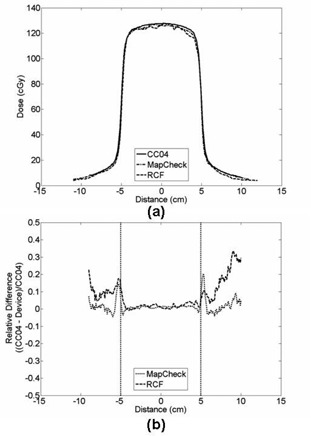
Cross‐plane profile (a) of $10\times 10\;{\text{cm}}^{2}$ field size at air gap of 0 cm and solid water slab of 20 cm for the MapCHECK, RCF, and CC04; (b) of dose difference for the cross‐plane profile from (a). The two dotted vertical lines present 50% penumbra point of the field.

Figure 5 shows the profiles of 20 cm solid water slab at 0 cm air gap for $10\times 10\;{\text{cm}}^{2}$ field size measurements. For this case, the CAX percent dose difference and the average difference for MapCHECK were −1.1% and 1.0% (1σ=1.3%), respectively. For the RCF, the CAX percent dose difference and the average difference were −1.0% and 2.9% (1σ=2.0%), respectively. Overall, for the dosimeter, the CAX percent dose difference and the average difference for the 0 cm air gap measurement for both solid water slabs thicknesses (i.e., 10 cm and 20 cm) ranged from −2.4% to −0.2% and −0.1% (1σ=2.0%) to 1.2% (1σ=2.6%), respectively. Likewise, the RCF showed that the CAX percent dose difference and the average difference ranged from −2.8% to −1.0% and 0.5% (1σ=1.9%) to 3.1%(1σ=2.6%), respectively. Table 1 provides a complete list of results for the 0 cm air gap setup.

**Table 1:** Central axis percent dose difference and average difference at 0 cm air gap. CC04 was used as the reference.

	*0 cm Air Gap*							
	*10 cm Solid Water Slab*				*20 cm Solid Water Slab*			
	*MapCHECK*		RCF		MapCHECK		*RCF*	
*Field Size*	*CAX*	*Average Difference (1σ)*	*CAX*	*Average Difference (1σ)*	*CAX*	*Average Difference (1σ)*	*CAX*	*Average Difference (1σ)*
$3\times 3\;{\text{cm}}^{2}$	−1.8%	1.2% (2.6%)	−1.6%	3.0% (2.1%)	−2.4%	1.1% (1.8%)	−2.0%	3.1% (2.6%)
$5\times 5\;{\text{cm}}^{2}$	−1.1%	1.0% (1.7%)	−2.8%	2.8% (1.4%)	−1.8%	1.2% (1.3%)	−2.7%	2.5% (1.5%)
$7\times 7\;{\text{cm}}^{2}$	−0.2%	−0.1% (2.0%)	−1.4%	0.5% (1.9%)	−1.6%	0.9% (1.5%)	−1.6%	2.4% (1.9%)
$10\times 10\;{\text{cm}}^{2}$	−0.5%	0.8% (3.2%)	−2.2%	1.8% (4.2%)	−1.1%	1.0% (1.3%)	−1.0%	2.9% (2.0%)

**Figure 6:**
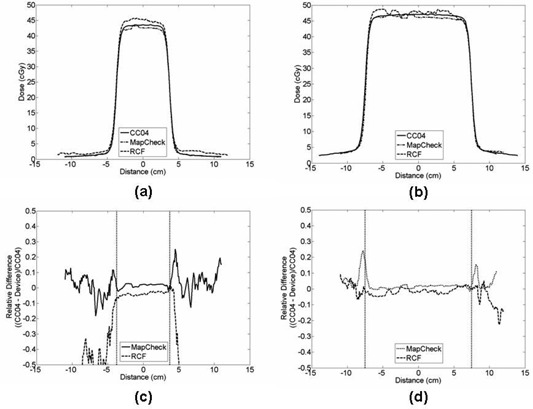
Dose profiles measured with a 40 cm air gap and 20 cm thick solid water slabs for field sizes of: (a) $5\times 5\;{\text{cm}}^{2}$ and (b) $10\times 10\;{\text{cm}}^{2}$. The dose difference profiles for (a) and (b) are shown in (c) and (d), respectively.

Figure 6 shows a profile for an air gap of 40 cm for MapCHECK, RCF, and CC04 at a solid water thickness of 20 cm. The CC04 was used as the reference. The CAX percent dose differences for the $5\times 5\;{\text{cm}}^{2}$ field size for MapCHECK and RCF were −2.2% (Table 2) and 4.1% (Table 3), respectively. For the average difference, the results were 1.4% (1σ=1.2%) and −3.8%(1σ=1.3%) for MapCHECK and RCF, respectively. For the $10\times 10\;{\text{cm}}^{2}$ profiles, the CAX percent dose difference for MapCHECK and RCF were −1.8% and 2.2%, respectively. For the average difference, the results were 1.7% (1σ=2.1%) and −1.5%(1σ=1.9%) for MapCHECK and RCF, respectively. Overall, the CAX percent dose difference and the average difference for

**Table 2:** Central axis percent dose difference and average difference between the MapCHECK and CC04 profiles.

	*30 cm Air Gap*				*40 cm Air Gap*			
	*10 cm Solid Water Slab*		20 cm Solid Water Slab		10 cm Solid Water Slab		*20 cm Solid Water Slab*	
*Field Size*	CAX	Average Difference (1σ)	CAX	Average Difference (1σ)	CAX	Average Difference (1σ)	CAX	*Average Difference(1σ)*
$3\times 3\;{\text{cm}}^{2}$	−1.3%	−0.1% (1.4%)	−1.7%	0.7% (1.6%)	−2.7%	1.5% (1.5%)	−2.4%	1.8% (1.2%)
$5\times 5\;{\text{cm}}^{2}$	−0.7%	0.3% (1.4%)	−1.4%	1.0% (1.3%)	−2.2%	1.5% (1.3%)	−2.2%	1.4% (1.2%)
$7\times 7\;{\text{cm}}^{2}$	−0.4%	0.5% (1.3%)	−1.3%	1.0% (1.4%)	−2.0%	1.9% (1.9)	−2.0%	2.1% (1.6%)
$10\times 10\;{\text{cm}}^{2}$	−0.0%	−0.2% (1.6%)	−0.7%	0.8% (1.3%)	−1.8%	1.7% (1.7%)	−1.8%	1.7% (2.1%)

	*50 cm Air Gap*				*60 cm Air Gap*			
	*10 cm Solid Water Slab*		20 cm Solid Water Slab		10 cm Solid Water Slab		*20 cm Solid Water Slab*	
*Field Size*	CAX	Average Difference (1σ)	CAX	Average Difference (1σ)	CAX	Average Difference (1σ)	CAX	*Average Difference(1σ)*
$3\times 3\;{\text{cm}}^{2}$	−2.0%	2.2% (2.0%)	−2.8%	2.5% (1.4%)	−3.6%	4.0% (1.7%)	−2.6%	2.6% (1.0%)
$5\times 5\;{\text{cm}}^{2}$	−1.3%	1.0% (1.3%)	−2.2%	1.2% (2.0%)	−3.1%	3.4% (1.1%)	−2.5%	2.7% (1.3%)
$7\times 7\;{\text{cm}}^{2}$	−1.1%	1.8% (3.3%)	−2.6%	2.1% (1.4%)	−3.0%	3.5% (1.7%)	−2.0%	2.3% (1.0%)
$10\times 10\;{\text{cm}}^{2}$	−1.9%	1.9% (2.3%)	−2.8%	2.2% (2.0%)	−3.4%	3.7% (2.4%)	−3.2%	2.7% (1.1%)

**Table 3:** Central axis difference and average difference between the RCF and CC04 profiles at the 40 cm air gap with a 20 cm solid water slab.

*Field Size*	*CAX*	*40 cm Air Gap Average Difference (**1**σ)*
$3\times 3\;{\text{cm}}^{2}$	1.9%	−0.2% (3.2%)
$5\times 5\;{\text{cm}}^{2}$	4.1%	−3.8% (1.3%)
$7\times 7\;{\text{cm}}^{2}$	2.7%	−1.7% (1.6%)
$10\times 10\;{\text{cm}}^{2}$	2.2%	−1.5% (1.9%)

the dosimeter for four air gaps (i.e., 30 cm, 40 cm, 50 cm, and 60 cm) and both solid water slab thicknesses (i.e., 10 cm and 20 cm) ranged from −3.6% to 0.0% and from −0.2% (1σ1.6%) to 4.0% (1σ=1.7%), respectively. For the RCF, the overall CAX percent dose difference and the average difference for the 40 cm air gap with 20 cm solid water slabs ranged from 1.9% to 4.1% and from −3.8% (1σ=1.3%) to −0.2% (1σ=3.2%), respectively. Tables 2 and 3 provide a complete list of the results for the four air gaps and two solid water slab thicknesses. Finally, Figure 7 shows a plot of the average difference with respect to air gaps for each field size for two solid water slabs. These plots reveal that as the air gaps increased, the MapCHECK agreement decreased slightly.

**Figure 7:**
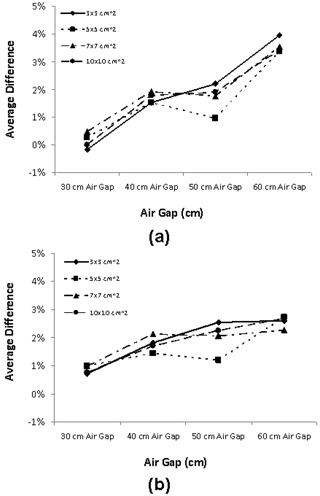
Average difference versus air gap: (a) for the 10 cm solid water slab; (b) for the 20 cm solid water slabs.

For the IMRT fields, the mean gamma index passing rate was 92.5% (minimum passing rate was 89.1% and maximum passing rate was 94.7%) at 0 cm air gap. For a 50 cm air gap, the mean passing rate was 91.9%, with a minimum and maximum passing rate of 87.6% and 97.2%, respectively (Table 4).

**Table 4:** Evaluation results of five IMRT fields using the gamma index comparing MapCHECK and RCF at an air gap of 50 cm with 10 cm of solid water slabs. The gamma index criteria were defined as 3% and 3 mm at the isocenter.

*Field Number*	*Passing Rate at 0 cm Air Gap (%)*	*Passing Rate at 50 cm Air Gap (%)*
1	94.7	94.6
2	89.1	97.2
3	92.8	87.6
4	93.6	87.7
5	92.3	92.3

### B. Characterizing scatter photons from the phantom to the detector

**Figure 8:**
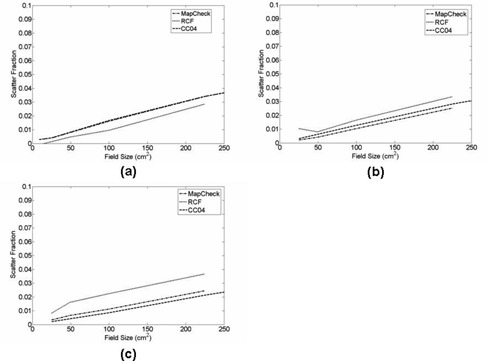
Central axis scatter fraction with field sizes of 3×3,5×5,7×7,10×10, and $15\times 15\;{\text{cm}}^{2}$ for air gap: (a) 40, (b) 50, and (c) 60 cm, with 20 cm of solid water slabs. For the central axis scatter fraction, MapCHECK, RCF, and CC04 were used for the evaluation.

**Figure 9:**
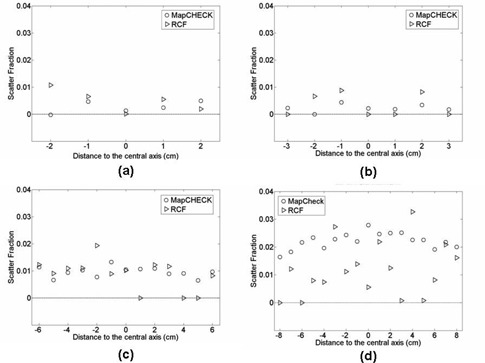
Scatter fraction profiles for the case of 50 cm air gap and 20 cm solid water slabs with field sizes of: (a) 3×3, (b) 5×5, (c) 10×10, and (d) $15\times 15\;{\text{cm}}^{2}$. The dotted horizontal lines indicate the zero percentage scatter fraction.

Figure 8 shows a plot of a phantom‐to‐detector scatter fraction at the CAX for three air gaps (i.e., 40 cm, 50 cm, and 60 cm). For all three dosimeters, the scatter fraction ranged from < 1% to < 3%. The general trend appeared to be that the scatter fraction increased as the field size increased. No significant differences occurred with increasing air gaps. Figure 9 shows the scatter fraction profile at the 50 cm air gap with the dosimeter and RCFs. Likewise, the scatter fraction profiles also increased as the field size increased. The scatter fraction profiles for air gaps of 40 and 60 cm showed a very similar trend to that at the 50 cm air gap.

### C. Scatter kernel

**Figure 10:**
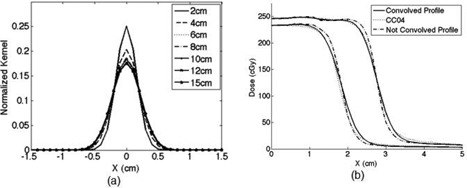
Normalized scatter kernels (a) at seven different depths for $5\times 5\;{\text{cm}}^{2}$ field size. Back‐projected beam profiles (b) for $3\times 3\;{\text{cm}}^{2}$ and $5\times 5\;{\text{cm}}^{2}$ field sizes before convolution (dot‐dashed line) and after convolution (solid line) with the scatter kernels. We also plotted ion chamber‐measured profiles (dotted line) at the same depth, for comparison.

(Figure 10a) shows a plot of scatter kernels for various depths for a $5\times 5\;{\text{cm}}^{2}$ field size, and Fig. 10(b) shows a profile of convolved dose profiles and ionization chamber profiles at the depth of 10 cm for a field size of $3\times 3\;{\text{cm}}^{2}$ and $5\times 5\;{\text{cm}}^{2}$. As depth increased, the scatter kernel tended to get broader and lower in amplitude (Fig. 10(a) because the increase in depth resulted in more laterally scattered photons.

### D. Evaluating dose reconstruction

**Figure 11:**
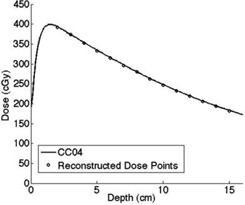
Comparison between the reconstructed depth‐dose and ion chamber‐scanned depth‐dose for a 6 MV photon beam with a field size of $5\times 5\;{\text{cm}}^{2}$. For the dose reconstruction, we used a source‐to‐detector distance of 160 cm and 20 cm solid water slabs for the measurement.

**Figure 12:**
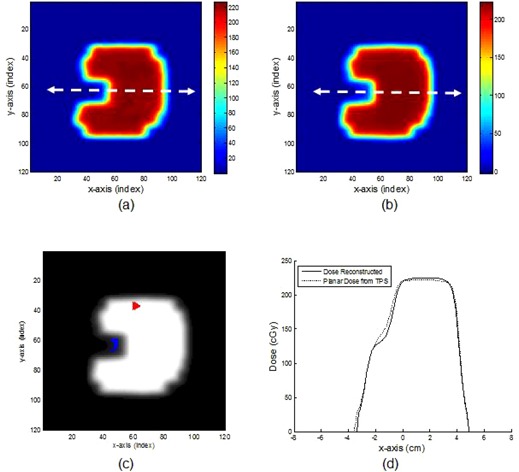
Comparison between the reconstructed (a) and TPS‐calculated (b) planar dose distributions for one of the six conformal fields. Six conformal field transit fluences were irradiated with a setup of 160 cm SDD with 20 cm solid water slab using 300 MU. The dose was reconstructed at a 10 cm depth. Gamma index result (c) (99.6% passing rate) with failure points is indicated. The red points are the hot spots and the blue points are the cold spots. Profile (d) of dose reconstructed and planar dose from the TPS (dotted arrow indicates the location of the profile taken).

**Figure 13:**
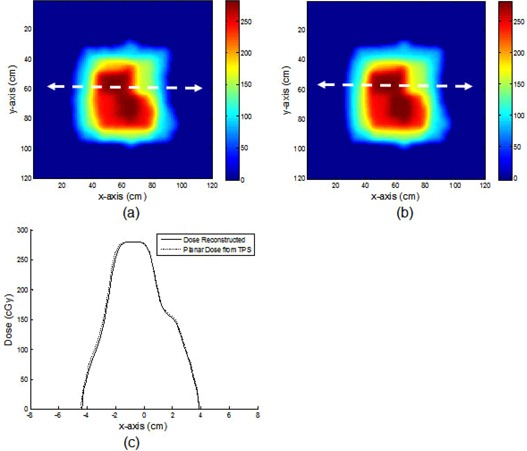
Comparison between the reconstructed (a) and TPS‐calculated (b) planar dose distributions for one of the five IMRT fields. The transit fluence of each segment was collected separately. Comparison (c) of the profiles taken at the position indicated by the dashed line.

Figure 11 shows a plot for a depth‐dose profile for a $5\times 5\;{\text{cm}}^{2}$ field size with 20 cm of solid water slabs. The discrepancy between the reconstructed dose and the CC04 was less than ± 1%. Both conformal and IMRT fields had very good gamma index passing rates. Of the six conformal fields, four had a gamma index passing rate of 100% and two had a gamma index passing rate of 99.4% and 99.6% (a conformal field is shown in Fig. 12). Of the five IMRT fields, three had a gamma index passing rate of 100% and two had gamma index passing rates of 99.6% and 98.8% (an IMRT field is shown in Fig. 13).

## IV. DISCUSSION

The transit dosimetry using the MapCHECK 2D array dosimeter produced very similar results to those reported by others using amorphous silicon EPID systems even though the device was not utilized in its original design. Kirby and Williams^(59)^ calibrated an EPID system with a silicon diode and were able to obtain an agreement within 3% on the CAX. Pasma et al.^(62)^ used a charge‐coupled device camera‐based EPID system to report a portal dose using a deconvolution algorithm method, which showed an agreement within 1% (1 SD) with ionization chamber measurements at a 40 cm air gap. Grein et al.^(57)^ did similar work using an amorphous silicon EPID system. They evaluated in‐plane and cross‐plane profiles of flat and wedge fields against Kodak XV films and an ionization chamber using 6 MV and 18 MV photon beams at a 150 cm SDD. Open and wedge field profiles measured with the EPID system showed agreement to a maximum of 5% and 8%, respectively, compared with the film. A comparison of relative transmission measurements between an EPID and an ionization chamber showed an agreement of 6% and 2% for 6 and 18 MV, respectively, for a solid water slab thickness of 21 cm and SDD > 130cm. Furthermore, Nijstan et al.^(74)^ described a global calibration model for amorphous silicon EPID for transit dosimetry. They reported mean relative dose difference of ‐8.2%± 17.9% (1SD) and 10.0%± 7.6% (1SD) for an asymmetric 10 cm×10 cm field and an irregular MLC field, respectively. Finally, Chen et al.^(56)^ reported a convolution model‐based calibration method for a flat amorphous silicon panel EPID system. For all fields except the smallest field centered about the CAX, they reported that the calibrated flat‐panel profiles matched the measured dose profiles with little or no systematic deviation and ~3% (2 SDs) accuracy for the in‐field region. The works presented by others have shown that EPID could perform as a dosimeter to within acceptable errors at a cost of significant image processing and corrections (e.g., deconvolution). From our study, the maximum average difference of 50 cm air gap for 20 cm solid water slab using the 2D array dosimeter with respect to the ionization chamber was 2.5% (see Table 2), which were consistent with the results from the EPID studies without the additional correction that was required with the EPID method. Thus, the 2D array dosimeter can be used to measure the transit fluence as accurately as the EPID method.

Although the accuracy of measuring a transit dose distribution using an EPID system has been established, other factors make using devices similar to the MapCHECK system more desirable for a transit dosimetry application. The single greatest problem with the EPID system is that it is an imaging device, not a dosimeter. Thus, converting the portal image to a portal dose distribution requires significant post‐detection correction, which is not required when using the MapCHECK. As mentioned above, Chen et al.^(56)^ achieved a highly accurate transit dose measurement with a flat amorphous silicon panel EPID system, but only by utilizing empirical convolution kernels to model the dose deposition in the EPID and in the water. Additionally, to account for the individual variations in the EPID pixel response, the flat‐panel signal was multiplied by a pixel‐dependent sensitivity factor. In a similar study, Warkentin et al.^(75)^ developed a convolution‐based calibration procedure to use an amorphous silicon flat‐panel EPID to accurately verify dosimetric IMRT fields. The results agreed to within 2.1% with those measured with film for open fields of $2\times 2\;{\text{cm}}^{2}$ and $10\times 10\;{\text{cm}}^{2}$.

The MapCHECK system has limitations, including a low spatial resolution, an insufficient detection area, and variation of agreements between CC04 and MapCHECK. The current arrangement of detector spacing does not provide enough dose points to sufficiently reconstruct the dose when it is applied at an extended SSD. For this reason, a motorized stepper is used to increase the detector resolution for this study. Also, the dosimeter's detection area is not sufficient for transit dosimetry. For proper clinical implementation, a 2D array dosimeter should be fabricated with a better resolution and a larger detection area (e.g., $40\times 40\;{\text{cm}}^{2}$). And lastly, standard deviations of average difference between MapCHECK and CC04 with respect to air gaps, field sizes, and solid water slab thicknesses range from 1.0% to 3.3% (see Table 2). This may be attributed to the array detector's inability to properly calibrate for the low‐energy photon spectrum. Further investigation is needed to fully characterize this phenomenon.

Proper characterization of scatter photons from the phantom down to the detector is an integral part of dose reconstruction for which the primary transit dose distribution was needed for backprojection dose reconstruction. Therefore, a comprehensive study was done to characterize the scatter fraction with respect to the air gaps and the field sizes. As seen in Fig. 8, the scatter fraction for all three air gaps (i.e., 40 cm, 50 cm, and 60 cm) increased almost linearly with increasing field sizes, a phenomenon that many investigators have previously reported.^(^ ^30^ ^,^ ^45^ ^,^ ^70^ ^,^ ^76^ ^)^ In this work, the scatter fraction was < 1% for small field sizes and increased to 3% for large field sizes. Similarly, Fig. 9 shows the scatter fraction profiles for air gap of 50 cm increased with increasing field sizes. The maximum scatter fraction for the smallest field size $\left(3\times 3\;{\text{cm}}^{2}\right)$ was < 2%. As the field size increased, the scatter fraction increased to ~ 3% at the largest field size $\left(15\times 15\;{\text{cm}}^{2}\right)$. This finding is consistent with what was reported by Swindell and Evans^(76)^ and Boellaard et al.^(30)^ In the Boellaard study, researchers used a homogeneous polystyrene phantom with EPID to determine the scatter contribution with respect to air gap. They showed that for large air gaps (> 50cm) the scattered dose increased linearly with field size but that the amount of scatter was < 2%. Swindell and Evans quantified the scatter contribution using Monte Carlo calculation with respect to air gaps. They showed the scatter contribution for air gaps > 50cm was < 5%. The scatter fraction determined in this work is consistent with that reported by other investigators, which showed that scatter fraction at large air gaps (>50 cm) was generally < 2% and negligible.

The gamma index passing rate for the five IMRT fields is consistent with the findings of Wendling et al.^(54)^ They delivered five clinical step‐and‐shoot IMRT plans to a 20 cm thick polystyrene slab phantom to the midplane. For each field, all the segments were collected separately and summed up to generate an intensity‐modulated dose distribution. The gamma index criteria for comparing the reconstructed dose using EPID and film measurements was 2%/2 mm. The percent gamma index passing rates for the five IMRT fields were 99.96%, 99.97%, 99.95%, 99.99%, and 99.86%. We found that *in vivo* backprojection dose reconstruction via transit dosimetry using a 2D array dosimeter is as accurate as using an EPID.

Collection of transit fluence using the MapCHECK 2D array dosimeter with the motorized XY stepper took about 45 minutes per field (six conformal fields) and/or segment (five segments for each IMRT field) because the dosimeter had to be translated in the X and Y axes to achieve a 2 mm grid spacing. The method proposed in this study would not be clinically acceptable because it would be not only impractical in a real clinical setting, but would also require multiple exposures. The ideal setup for a 2D array dosimeter is a $40\times 40\;{\text{cm}}^{2}$ active area with high spatial resolution, and with enough buildup layers to establish charge particle equilibrium.

With such favorable agreement between the reconstructed dose, TPS‐generated planar dose, and RCFs, our proposed backprojection dose‐reconstruction method could possibly be used for *in vivo* interfractional verification. This application will not only evaluate the dose deposition within the patient, but it also has the potential to evaluate the positional and geometrical accuracy of a target and/or critical structures. When used in conjunction with image‐guided radiotherapy, this method can greatly improve treatment validation.

The proposed *in vivo* dose reconstruction for treatment validation could be further refined from a 2D planar dose reconstruction to a 3D dose reconstruction using a volumetric image dataset (cone‐beam CT) acquired on the day of the treatment. As the treatment is delivered to the patient, the transit fluences from all the fields are collected using a high‐resolution dosimeter located under the patient (SDD ≥ 150cm). Once collected, they are used to reconstruct a 3D dose distribution delivered to the patient using the dose calculation algorithm described in this study. Transit fluences are used to reconstruct the dose delivered to the target by ray‐tracing the detected dose through the volumetric image for appropriate gantry angles. In addition to volumetric dose reconstruction, structure contours could be imported from the TPS to compute the dose‐volume histogram for comparison. This technique would provide the clinician the tools to monitor the dose delivered to the patient on a fractional and/or cumulative basis.

## V. CONCLUSIONS

The purpose of this study was to demonstrate the feasibility of using a 2D array dosimeter for *in vivo* dose reconstruction via transit dosimetry. The 2D array dosimeter used for the feasibility study was MapCHECK. The accuracy of the transit fluence measurement study showed that the MapCHECK system had an accuracy ranging from −3.6%−0.0% at central axis, depending on the air gap used. The agreement between the 2D array dosimeter and the ionization chamber and RCF profiles for the transit dose distribution measurement ranged from ‐3.8%~ 2.1%. The scatter contribution from the phantom to the 2D array dosimeter was ≤ 3% for the air gaps considered. For the preliminary study, the gamma index passing rates for the six conformal fields were very good. Out of the six fields, four fields showed a 100% gamma index passing rate, while the remaining two showed 99.4% and 99.6% gamma index passing rates. For the five IMRT fields, three showed a 100% gamma index passing rate, while the remaining two showed 99.6% and 98.8% gamma index passing rates. Lastly, the current form of the array detector system may not be appropriate for clinical implementation due to its low spatial resolution and small detection area. With much higher resolution and larger detection area, the array detector system should be able to function both as a dosimeter and an imager.
